# Symptomatic and Regional Therapeutics

**Published:** 1910-12

**Authors:** 


**Symptomatic and Regional Therapeutics.** By **George Howard Hoxie,** M.D., Professor of Internal Medicine in the School of Medicine, University of Kansas, Lawrence. Octavo, pp. 517. Illustrated. New York and London: D. Appleton & Co. 1910. (Cloth, $4.00.)

This is one of the better books of the year. It was built to follow the curriculum adopted by the council of education of the American Medical Association and it also follows the lines recommended by the committee in charge of the Carnegie Foundation for the Advancement of Teaching. The first part of the book is devoted to a consideration of symptoms and their relief, and of the relations of symptoms to pathological processes. In the second chapter the treatment of diseases is discussed with relation to the therapy of inflammations. Lastly, in a general review of the pathology, region by region, the application of the principles already laid down is made to the various diseases taken up in previous chapters. In an appendix (Part III) notes and memoranda on the drugs mentioned have been added, to save the reader the need of referring to some materia medica for dosage and official preparations. The demand for this book should be great. J. A. R.

